# Synergistic Flame Retardancy of Phosphatized Sesbania Gum/Ammonium Polyphosphate on Polylactic Acid

**DOI:** 10.3390/molecules27154748

**Published:** 2022-07-25

**Authors:** Qing Zhang, Huiyuan Liu, Junxia Guan, Xiaochun Yang, Baojing Luo

**Affiliations:** College of Chemistry, Tangshan Normal University, Tangshan 063000, China; huiyuanliu123@sina.com (H.L.); gjxia1103@126.com (J.G.); yangxiaochun313@163.com (X.Y.); xiaoluobo_19881116@163.com (B.L.)

**Keywords:** polylactic acid, sesbania gum, ammonium polyphosphate, flame retardancy

## Abstract

Phosphating sesbania gum (DESG) was obtained by modifying sesbania gum (SG) with 9,10-dihydro-9-oxa-10-phosphaphenanthrene-10-oxide (DOPO) and endic anhydride (EA). The structure of DESG was determined using Fourier transform infrared (FTIR) spectroscopy and nuclear magnetic resonance spectroscopy (^1^H-NMR). Flame-retardant polylactic acid (PLA) composites were prepared by melt-blending PLA with DESG, which acted as a carbon source, and ammonium polyphosphate (APP), which acted as an acid source and a gas source. The flame retardancy of the PLA composite was investigated using vertical combustion (UL-94), the limiting oxygen index (LOI) and the cone calorimeter (CONE) test. Thermal properties and morphology were characterized via thermogravimetric analysis (TGA) and field emission scanning electron microscopy (FESEM), respectively. Experimental results indicated that when the mass ratio of DESG/APP was equal to 12/8 the LOI value was 32.2%; a vertical burning test (UL-94) V-0 rating was achieved. Meanwhile, the sample showed a lowest total heat release (THR) value of 52.7 MJ/m^2^, which is a 32.5% reduction compared to that of neat PLA. Using FESEM, the uniform distribution of DESG and APP in the PLA matrix was observed. The synergistic effect of DESG and APP effectively enhanced the flame retardancy of PLA. Additionally, the synergistic mechanism of DESG and APP in PLA was proposed.

## 1. Introduction

The impact of worldwide production and consumption of petroleum-based plastics on economic and ecological sustainability has attracted widespread attention; these crises can be alleviated by developing bio-based plastics [1,2,3]. As an important environmentally friendly bio-based polymer, polylactic acid (PLA) can be obtained from corn, wheat, sugar beet, etc.; it completely degrades to carbon dioxide and water in a composting environment [4,5,6].

Recently, PLA materials have been commercialized in the fields of packaging materials, textile materials and medical materials because of their multiple advantages over traditional petro-polymers, including excellent mechanical properties, transparency, biocompatibility, biodegradability and processability [7,8,9,10]. However, the limiting oxygen index (LOI) of PLA is only 19%, which makes it easy to burn; a large number of molten droplets are formed during the burning process, which significantly restricts its commercial applications on a large scale [11,12].

Various flame-retardant additives have been incorporated into PLA matrices to solve this problem [13,14,15,16,17,18]. Polyphosphates, such as ammonium polyphosphate (APP), melamine polyphosphate, tris (hydroxymethyl)-aminomethane polyphosphate and isosorbide-based polyphosphonate, were introduced to PLA as halogen-free flame-retardants to increase its flame retardancy [19,20,21,22]; APP was often used in intumescent flame-retardants (IFR). As an eco-friendly flame-retardant, IFRs exhibit excellent flame retardancy on PLA due to their high effectiveness, low smoke production, low toxicity and low corrosiveness [23,24]. However, the char formation of pentaerythritol that usually acts as a carbon source in traditional IFRs is poor; a large amount of IFR needs to be added to achieve a vertical burning test (UL-94) V-0 rating, which leads to a significant decrease in the mechanical properties of PLA materials. Therefore, finding a carbon source with high flame-retardant efficiency is the key to improving the flame-retardant efficiency of IFRs in PLA.

With the development of bio-based materials and the increasing demand for ecologically sustainable development, research on bio-based flame-retardants has attracted the attention of many researchers. Bio-based polysaccharides such as starch, cellulose, lignin, cyclodextrin and chitosan have been used as “green” carbon sources in the flame-retardant modification of PLA [25,26,27,28,29]. Sesbania gum (SG) is a natural polysaccharide found in the seeds of sesbania, a native plant of China. It is a galactomannan; its main structure consists of one galactose on every other mannose unit. SG is an attractive biopolymer because of its abundance, low cost, biodegradability and potential application in the production of biodegradable films [30]. The large number of hydroxyl groups on the surface of these polysaccharides resulting in obvious phase separation from a hydrophobic matrix (such as PLA), resulting in poor interfacial compatibility between them and PLA. DOPO is commonly used as a reactive flame-retardant with high thermal stability, good antioxidant property, excellent flame-retardant ability and environmental compatibility. The reactive P–H bond in its structure may interact with unsaturated double bonds. Endic anhydride (EA), bicyclo[2.2.1]hept-5-ene-2,3-dicarboxylic anhydride, also has high reactivity, containing both unsaturated double bonds and anhydride bonds. The P–H bond of DOPO can undergo an addition reaction with the double bond of EA; the resulting product is further covalently bonded to the side chain of SG to reduce the polarity of SG and increase its compatibility with PLA. In the present work, SG was modified with DOPO and EA to prepare phosphorylated sesbania gum (DESG). Ammonium polyphosphate (APP), an acid source and gas source in IFRs, was combined with DESG and then added to PLA, resulting in a PLA-based composite material with flame retardancy. The synergistic flame retardancy of DESG and APP on PLA was investigated, thermal properties and morphologies of the resulting composites were characterized and the flame-retardant mechanism of DESG and APP in PLA was proposed.

## 2. Results

### 2.1. Structural Characterization of DESG

**Figure 1:**
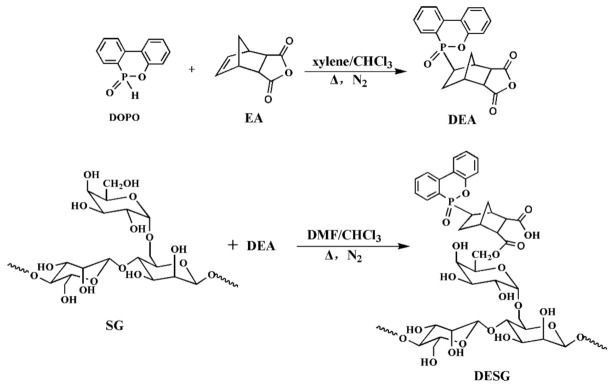
A DESG preparation route.

The preparation process of DESG consisted of two steps. In the first step, the P–H bond of DOPO and the double bond of EA underwent an addition reaction to prepare phosphatized EA (DEA). In the second step, the hydroxyl group of SG reacted with the anhydride group of DEA to obtain DESG. This process is shown in Figure 1.

**Figure 2:**
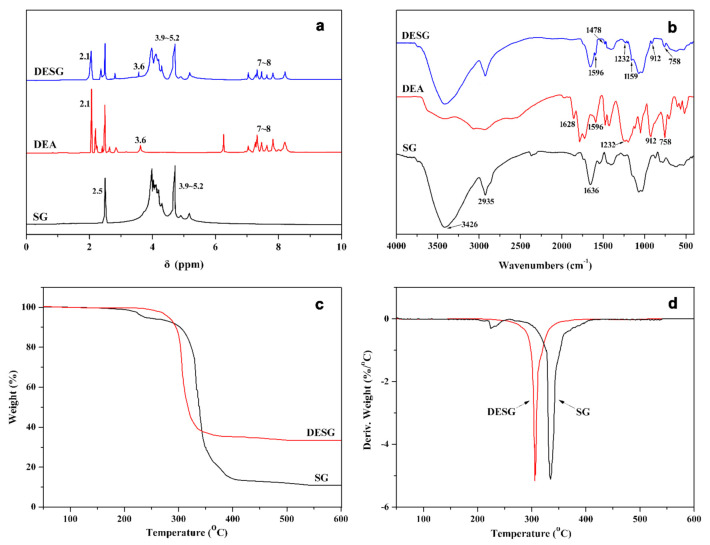
SG and DESG curves: (**a**) ^1^HNMR, (**b**) FTIR, (**c**) TGA and (**d**) DTG.

The structures of SG, DEA and DESG were characterized using FTIR spectroscopy and ^1^H-NMR. Figure 2a,b show the ^1^H-NMR and FTIR spectra of SG, DEA and DESG, respectively. Compared with those of DOPO [31] and EA [32], in the ^1^H-NMR spectrum of DEA the P–H signal (at δ 8.82 ppm in DOPO) and the peak (at approximately δ 6.5 ppm) attributed to the double bond of EA disappeared. However, new absorption peaks located at δ 3.6 and 2.1~2.8 ppm appeared, which demonstrated the occurrence of an addition reaction between DOPO and EA. For DESG, the signal that appeared in the range of δ 3.9~5.2 ppm was from SG; peaks at approximately δ 7~8 ppm could be assigned to the biphenyl group in DOPO, indicating that DEA was successfully grafted onto SG. All of this can also be confirmed in the FTIR spectra of DEA and DESG. For DEA, the stretching vibration band of double bond at 1628 cm^−1^ and the characteristic peak of P–H at 2436 cm^−1^ vanished. In the FTIR spectrum of DESG, the absorption peak of the anhydride group at 1864 cm^−1^ disappeared, but the absorption peaks of P–O–Ph (1159, 912, 758 cm^−1^), P=O (1232, 1208 cm^−1^) and P–Ph (1596, 1478 cm^−1^) [33,34] were still present.

The TGA and DTG curves of SG and DESG are shown in Figure 2c. Thermal stabilities were determined from the temperature at 5% weight loss, which was defined as the onset degradation temperature. From Figure 2c, we can see that the weight loss of SG occurred in two steps, including dehydration and thermolysis processes. The onset degradation temperature of SG was approximately 237.6 °C. DESG underwent only one stage of thermal degradation due to the thermolysis of the polysaccharide component. The onset degradation temperature shifted to a higher value (282.5 °C) compared with that of SG, which indicated that the introduction of the phosphorus element improved the early thermal stability of DESG. However, it can be found from the DTG curve that the maximum degradation rate temperature of DESG dropped approximately 28 °C. This was because the high temperature broke down the phospholipid bonds and the resulting acidic substances promoted the decomposition of SG, leading to a decrease in the maximum decomposition temperature. The final char residue at 800 °C was 33.5%, which was much higher than that of SG (11.0%); therefore, the high char residue and speed of carbon formation make DESG a potentially excellent carbon source for an IFR.

### 2.2. Flame Retardant Properties of DESG/APP on Polylactic Acid

DESG was combined with APP and then added to PLA, resulting in PLA-based composites with flame retardancy. Here, DESG was used as a carbon source and APP acted as both an acid source and a gas source. The flame retardancy of PLA composites was measured using UL-94 and LOI. The results are given in Table 1. The neat PLA sample showed a LOI value of 19.5%. For binary composites with 20 wt% SG or DESG added to PLA alone, the LOI did not change significantly. Similarly, SG or DESG alone were not helpful in upgrading the UL rating for PLA substrate. The incorporation of APP alone improved the LOI value of the A20 binary composite to 26.2%. However, the molten droplets produced ignited the degreasing cotton; the composite achieved only a UL V-2 rating. For a ternary composite, A12S8, containing 12 wt% APP and 8 wt% SG, the LOI value increased to 29.6%, but the UL rating was still V-2. This indicated that the synergistic flame retardancy of nonphosphatized SG and APP on PLA was weak. While 12 wt% APP and 8 wt% DESG were introduced into PLA, the A12D8 composite achieved a LOI value of 32.2% and a V-0 rating. The improvement in flame-retardant properties was due to the synergy effect between APP and DESG. As a result of this synergy, adding only 10 wt% flame-retardant, like A7D3 or A5D5, made the composite reach the V-0 rating. The effect of the addition ratio of APP and DESG on the flame-retardant properties of PLA composites was also investigated. The results from Table 1 show that the LOI value of the composites increased gradually and then decreased with an increase in APP content.

**Figure 3:**
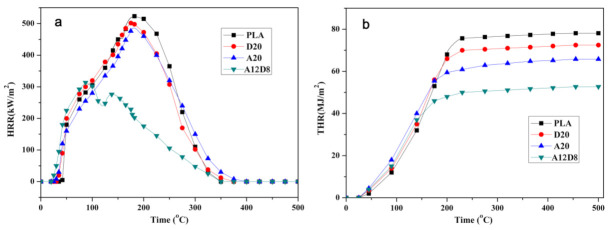
PLA, D20, A20 and A12D8 curves: (**a**) HRR and (**b**) THR.

In order to further investigate the synergistic effect of DESG and APP on PLA, a cone calorimeter (CONE) test, which is used to simulate the burning behavior of materials in a real fire, was conducted. The heat release rate (HRR) and total heat release (THR) curves of PLA and PLA composites are shown in Figure 3. In Figure 3a, one can see that neat PLA presented the largest peak heat release rate (pHRR) of 523.5 kW/m^2^. The pHRR value of D20 and A20 were 501.6 and 482.5 kW/m^2^, respectively. This indicated that the addition of DESG or APP alone cannot effectively reduce the HRR value. However, the pHRR value of the A12D8 sample decreased to 341.6 kW/m^2^, exhibiting a 34.7% decrease compared to that of neat PLA. The HRR curve of A12D8 showed a double peak, while only a single peak was observed for other samples. This bimodal phenomenon was characteristic of the intumescent flame-retardants [29,35]. As shown in the THR curves of PLA and PLA composites (Figure 3b), the THR values of neat PLA, D20 and A20 were 78.1, 72.4 and 65.8 MJ/m^2^, respectively. Similarly, although adding DESG or APP alone reduced the THR value compared with PLA, it was not as effective as adding both. The A12D8 sample showed the lowest THR value, 52.7 MJ/m^2^, which was a 32.5% reduction compared to that of neat PLA.

Compared with the results reported for polysaccharide-added PLA flame-retardant composites [26,36], A12D8 composites achieved a higher LOI value as well as lower pHRR and THR values. For A12D8 composites, a V-0 rating can be achieved by adding only 10 wt% flame-retardants; however, 15–25 wt% was added in some studies [28,35,37].

### 2.3. Thermal Stabilities of Flame-Retardant PLA Composites

**Figure 4:**
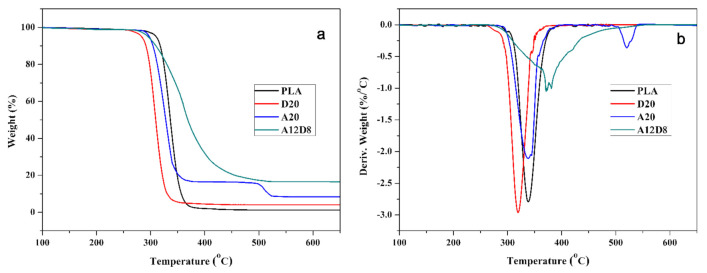
PLA, D20, A20 and A12D8 thermograms: (**a**) TGA and (**b**) DTG.

TGA was employed to evaluate the thermal stabilities of flame-retardant PLA composites. TGA and DTA thermograms of PLA and flame-retardant PLA composites in a nitrogen atmosphere (from 100 to 650 °C) are illustrated in Figure 4a,b. The initial decomposition temperature (T_i_), defined as the 5% weight loss temperature, the temperature of maximal weight loss rate (T_max_) and char yields at 400 and 600 °C are summarized in Table 2. For PLA, T_i_ and T_max_ were 309.4 °C and 338.7 °C, respectively. At approximately 400 °C, its decomposition was essentially complete; the char yield after 400 °C under N_2_ was only 0.3%. As a result of SG’s low decomposition temperature, the T_i_ of D20 decreased to 282.5 °C, with a char yield of 4.1% at 600 °C. The T_i_ of A20 was 296.5 °C and its char yield significantly increased at 400 °C. This occurred because the APP was decomposed in advance to generate phosphoric acid or polyphosphoric acid, which catalyzed the char formation of PLA itself. However, the char layer was unstable at high temperatures and underwent secondary decomposition at approximately 500 °C. For the A12D8 sample, thermal stability was higher after 300 °C than it was when adding APP or DESG alone; the presence of APP improved the thermal stability of DESG at a low temperature. Moreover, the weight loss of DESG at a low temperature was reduced compared with other samples. The char yield of A12D8 was as high as 32.1% and 16.8% at 400 °C and 600 °C, respectively. These results demonstrated the existence of the synergistic effect of APP and DESG combined in promoting char formation.

### 2.4. Dispersion of DESG/APP Flame-Retardant in PLA Matrix

**Figure 5:**
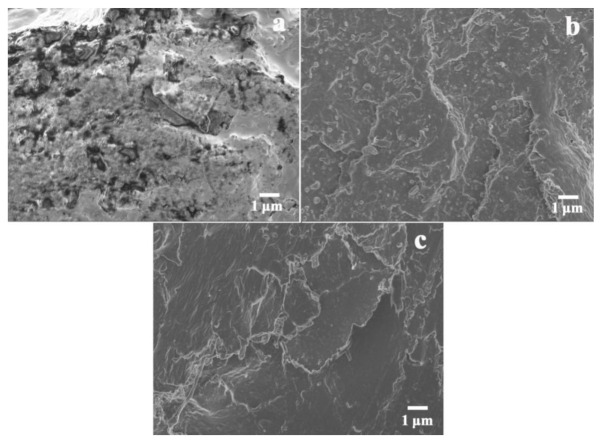
FESEM micrographs of PLA composites: (**a**) S20, (**b**) A20 and (**c**) A12D8.

To investigate the dispersion of DESG/APP flame-retardants in the PLA matrix, FESEM images of the fracture surfaces of D20, A20 and A12D8 samples are shown in Figure 5. It can be seen in Figure 5a,b that the fracture surfaces of the S20 and A20 samples were rough; there was an obvious phase interface between the SG or APP particles and the PLA matrix, indicating poor compatibility between the dispersed phase and the matrix. On one hand, because APP and SG are hydrophilic, there was a polarity difference between them and the hydrophobic PLA matrix. On the other hand, both APP and SG have strong intermolecular forces and are prone to agglomeration, which resulted in their poor dispersibility in PLA. After SG was phosphorylated by DEA, the hydrophobicity of SG improved significantly. Additionally, the carboxyl groups still existing on DESG could undergo esterification with the terminal hydroxyl groups of PLA during processing, which further improved the compatibility of DESG and PLA [38,39]. Therefore, homogeneous dispersion was found in the FESEM micrograph of the A12D8 sample (Figure 5c). In addition, the introduction of DESG also enhanced the compatibility of APP with the PLA matrix. This may be due to the reaction between the hydroxyl groups on DESG and the carboxyl groups on APP, which promoted better dispersion of the flame-retardant in the PLA matrix and further improved the synergistic flame-retardant efficiency of the flame-retardants.

It can be seen from the tensile properties of PLA and its composites (as shown in Table 3), that adding 20% DESG (sample D20) or APP (sample A20) alone significantly reduced the tensile strength and Young’s modulus of the composites, which ranged from 67.6 to 48.1 MPa and from 1.75 to 1.08 GPa, respectively. This indicated that the interfacial compatibility between DESG or APP and PLA was very poor, resulting in a serious decline in the mechanical properties of composites. For A20, the elongation at break increased slightly, which was attributed to the dilution effect of APP on the PLA molecular chain [19,40]. After adding both DESG and APP (sample A12D8), the decrease in the strength and modulus of the composites was effectively alleviated; it was speculated that the incorporation of DESG could improve compatibility between the dispersed phase and the PLA matrix and reduce the influence on the mechanical properties of the composites.

### 2.5. Flame-Retardant Mechanism

**Figure 6:**
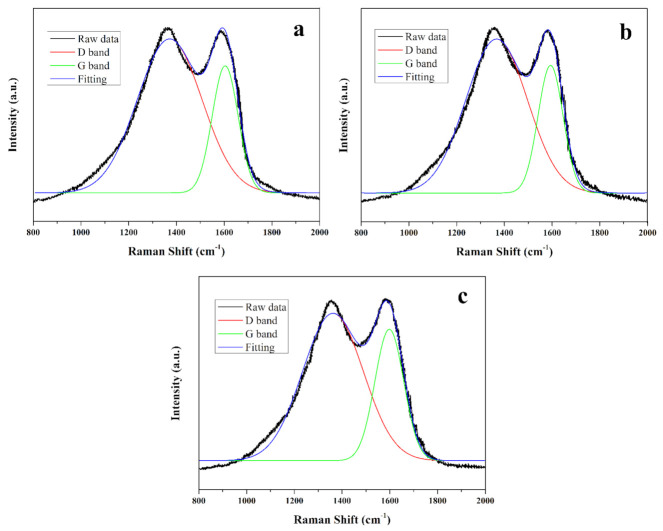
Raman spectra of (**a**) D20, (**b**) A20 and (**c**) A12D8 after cone calorimeter test.

To understand the flame-retardant mechanism in the condensed phase, the Raman spectra of char residues of D20, A20 and A12D8 samples after cone calorimeter tests were investigated; the results are shown in Figure 6. The vibrations of disordered carbon and graphitic carbon were detected near 1340 cm^−1^ and 1585 cm^−1^, which correspond to the D and G bands, respectively. The degree of graphitization was assessed using the value of the area ratio of D to G bands (AD/AG); a lower value suggested a higher graphitization degree of char layer, better shielding effect and higher thermal stability [41,42]. The AD/AG values of D20, A20 and A12D8 samples were 3.1, 2.9 and 2.3, respectively. The A12D8 sample had a lower AD/AG value compared to the others, indicating that the addition of both DESG and APP facilitated forming more graphitized char residue with a shielding effect for PLA.

**Figure 7:**
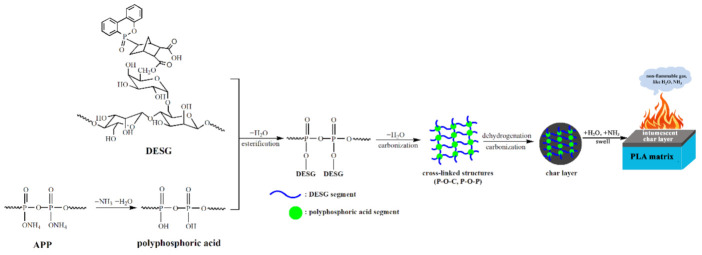
A possible synergistic mechanism of DESG/APP flame-retardant.

Figure 7 presents a possible synergistic mechanism of the DESG/APP flame-retardant. When the composite material was heated and burned, APP, which was an acid source and a gas source, first decomposed to generate ammonia and polyphosphoric acid (or polymetaphosphoric acid). Ammonia, a non-flammable gas, could have a diluting effect in the gas phase. An esterification reaction between hydroxyl groups in the DESG molecules and polyphosphoric acid might occur. Meanwhile, the polyphosphoric acid, with strong dehydration, can accelerate the carbonization of the ester to form a phosphorus–carbon char layer containing cross-linked structures of P–O–C and P–O–P. With a continuous increase in the external temperature, the cross-linked structures then underwent various chemical reactions such as dehydrogenation, carbonization, breakage of the chemical bond, etc., to form char residues rich in phosphorus and carbon. These char residues appeared macroscopically as black carbonaceous skeletons. The water vapor generated by the reaction and some incombustible gases (such as NH_3_) swelled the char layer, forming an intumescent protective layer. This intumescent layer, with high strength, good compactness and excellent thermal stability, covered the surface of the PLA matrix to produce a fine shielding effect, which further delayed the degradation rate of the material through effective thermal insulation and oxygen insulation and improved the flame-retardant performance. It is noteworthy that the esterification of DESG and polyphosphoric acid caused the initial weight loss of the composite, but at the same time a more stable carbon layer formed. This also explains why A12D8 had a 5% weight loss at a lower temperature of 273.0 °C, but less weight loss and a higher char residue at a higher temperature.

## 3. Materials and Methods

### 3.1. Materials

PLA (4032D) and SG were commercially obtained from Nature Work Company (Blair, NE, USA) and Ningbo Global Biomaterials Co., Ltd. (Ningbo, China), respectively. APP (analytical reagent) (AR) and DOPO (AR) were purchased from Sigma-Aldrich (Saint Louis, MO, USA). EA (AR), 4-dimethylaminopyridine (4DMAP) (AR), xylene (AR), chloroform (AR), anhydrous ethanol (AR) and N, N-Dimethylformamide (DMF) (AR), were supplied by Aladdin Industrial Co. (Beijing, China). All reagents were used without any further purification.

### 3.2. Preparation Procedures

#### 3.2.1. Preparation of DESG

A total of 50 g of DOPO was dissolved in 100 mL of xylene at 75 °C in a three-neck flask equipped with a condenser; 46 g of EA dissolved in 80 mL of chloroform was added to this solution under a nitrogen atmosphere, followed by a reflux with magnetic stirring for 24 h. After the reaction was completed and cooled to room temperature, the solvent was removed via rotary evaporation at 75 °C to obtain DEA with a yield of 91.8%.

A total of 30 g of SG and 100 mL of DMF were added to a three-necked flask; the solution was heated to 90 °C, followed by a reflux for 4 h under a nitrogen atmosphere. This mixture was cooled to 75 °C. Next, 30 g of DEA dissolved in chloroform and an appropriate amount of 4DMAP was added. The reflux reaction was continued for 24 h under nitrogen protection. After the reaction was completed, the mixture was cooled to room temperature, washed with anhydrous ethanol 5 times and vacuum dried at 75 °C for 10 h to obtain DESG with a yield of 83.5%.

#### 3.2.2. Blend Preparation of Flame-Retardant PLA Composites

PLA, APP, SG and DESG were dried in a vacuum drying oven at 80 °C for 24 h before use. The dried raw materials were weighed and premixed in a high-speed mixer for 8 min according to the formulations listed in Table 1. Next, the premixed blends were added to the mixing chamber of a torque rheometer to be melt-blended for 10 min at a temperature of 185 °C and a rotational speed of 45 rpm. The blends were compressed into plate specimens by a plate vulcanizer under 10 MPa for 5 min at 180 °C. Finally, these plates were cut and polished into standard splines for testing.

### 3.3. Analysis

The 1H-NMR spectra were measured on a Bruker AV II-400 MHz spectrometer (USA) using DMSO-d_6_ as a solvent at 25 °C. The FTIR spectra were recorded on a Nicolet is 50 FTIR spectrometer within the 4000–400 cm^−1^ region with KBr matrix.

The morphology of the fractured surfaces of specimens was observed with a ZEISS Sigma 300 FESEM (Schnelldorf, Germany) with an accelerating voltage of 10 kV.

TGA and DTG analyses were carried out with a PerkinElmer TGA 4000 thermogravimetric analyzer (Waltham, MA, USA) at a heating rate of 10 °C/min from 30 to 650 °C under a nitrogen atmosphere.

LOI values were recorded on a Jiangning JF-3 oxygen index analyser (Nanjing, China) with sample dimensions of 130 mm × 6.5 mm × 3 mm according to ASTMD 2863-97. UL-94 vertical burning tests were performed using a Jiangning CZF-3 horizontal and vertical combustion tester (Najing, China) with sample dimensions of 130 mm × 13 mm × 3.0 mm in accordance with ASTMD 3801-2010.

CONE tests were carried out with a FTT cone calorimeter (East Grinstead, England) under an external heat flux of 50 kW/m^2^. Sample dimensions were 100 mm × 100 mm × 3 mm according to ISO 5660.

Tensile properties were determined using a CMT4202 universal testing machine (Shenzhen, China) according to ASTM D638, at a test speed of 10 mm/min. Specimens were dumbbell-shaped with dimensions of 75 mm × 4 mm ×1 mm.

## 4. Conclusions

DESG was prepared by grafting SG with DOPO and EA. Flame-retardant PLA composites were obtained by melt-blending PLA with DESG and APP. Here, DESG acted as a carbon source due to its polysaccharide structure, while APP acted as an acid source and a gas source to catalyze the formation of char residue and release nonflammable gases, respectively. The flame retardancy, thermal properties, morphology and tensile properties of flame-retardant PLA composites were investigated. The results demonstrated that the synergistic effect of DESG and APP promoted the formation of an intumescent protective layer in PLA composites at a lower temperature, improved the high temperature stability of the composites and effectively enhanced the flame retardancy of PLA. When the mass ratio of DESG/APP is equal to 12/8, an LOI value of 32.2% and a UL-94 V-0 rating were achieved. A V-0 rating can also be reached at a lower addition ratio. Moreover, the addition of DESG could improve compatibility between the dispersed phase and the PLA matrix, as well as reduce the influence of the flame-retardant on the mechanical properties.

## Figures and Tables

**Table 1:** Formulations and flammability of PLA and flame-retardant PLA composites.

Samples	PLA (wt%)	APP (wt%)	SG (wt%)	EDSG (wt%)	LOI (%)	UL-94	Cotton Ignition
PLA	100	0	0	0	19.5	NR ^a^	Yes
S20	80	0	20	0	19.4	NR	Yes
D20	80	0	0	20	19.9	NR	Yes
A20	80	20	0	0	26.2	V-2	Yes
A12S8	80	12	8	0	29.6	V-2	Yes
A18D2	80	18	0	2	31.3	V-1	No
A15D5	80	15	0	5	31.9	V-0	No
A12D8	80	12	0	8	32.2	V-0	No
A9D11	80	9	0	11	29.2	V-0	No
A5D15	90	5	0	15	28.6	V-1	No
A9D1	90	9	0	1	25.8	V-1	No
A7D3	90	7	0	3	27.6	V-0	No
A5D5	90	5	0	5	26.1	V-0	No
A3D7	90	3	0	7	24.3	V-2	No

^a^ NR = no rating.

**Table 2:** TGA and DTG data for PLA, D20, A20 and A12D8.

Samples	T_i_ (°C)	T_max_ (°C)	Char Yield (%)	
			at 400 °C	at 600 °C
PLA	309.4	338.7	0.3	0.3
D20	282.5	319.2	4.2	4.1
A20	296.5	337.1	16.5	8.6
A12D8	273.0	343.6	32.1	16.8

**Table 3:** Tensile properties of PLA and its composites.

Samples	Tensile Strength (MPa)	Young’s Modulus (GPa)	Elongation at Break (%)
PLA	67.6	1.75	7.6
D20	52.3	1.35	6.2
A20	48.1	1.08	8.2
A12D8	63.4	1.63	7.3

## Data Availability

Not applicable.

## Sample Availability

Samples of the compounds are not available from the authors.
